# Supplementary material for: Development of chemokine network inhibitors using combinatorial saturation mutagenesis
Source: Commun Biol. 2025 Apr 3;8:549. doi: 10.1038/s42003-025-07778-6 (PMC11969024; doi:10.1038/s42003-025-07778-6)
Supplement: Supplementary file 4 — Supplementary Data 2 [file 42003_2025_7778_MOESM4_ESM.zip › SupplementaryData3/scripts/xNNKi.tar!/./script/analysis/JK/boxplots/HD2X_selectedCC_boxplot.pdf]

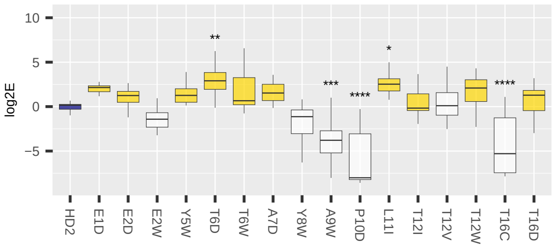

log2E
10
5
0
−5
**
*
***
****
****
HD2
E1D
E2D
E2W
Y5W
T6D
T6W
A7D
Y8W
A9W
P10D
L11I
T12I
T12V
T12W
T16C
T16D
